# New Staging System and Prognostic Model for Malignant Phyllodes Tumor Patients without Distant Metastasis: A Development and Validation Study

**DOI:** 10.3390/jcm12051889

**Published:** 2023-02-27

**Authors:** Zhaohui Ruan, Qi Quan, Qianyu Wang, Jiaxin Jiang, Roujun Peng

**Affiliations:** 1 Department of VIP Section, Sun Yat-sen University Cancer Center, State Key Laboratory Oncology in South China, Collaborative Innovation Center of Cancer Medicine, Guangzhou 510060, China; 2 Changping Laboratory, Beijing 102206, China

**Keywords:** malignant phyllodes tumor of the breast, breast cancer, cancer stage, prognostic model, machine learning

## Abstract

**Simple Summary:**

Malignant phyllodes tumor of the breast (MPTB) is a rare fibroepithelial tumor. Because of its rarity, research based on large clinical datasets is currently lacking. Moreover, the prognostic factors of MPTB have yet to be determined. Prognostic models and staging systems for MPTB patients are needed, but are also lacking. Here, we conducted a comparison between MPTB cases and invasive ductal carcinoma cases. Our findings reveal substantial differences in the clinical features of invasive ductal carcinoma and MPTB. We applied the KAPS algorithm to explore and establish a new stage- and age-stratification system. The system exhibited a good prognostic stratification ability for both the internal cohort and the external cohort. Furthermore, we developed independent prognostic models for MPTB using Cox proportional hazards regression and random survival forests (RSF). Finally, we built a user-friendly web app to allow researchers and doctors to access our model.

**Abstract:**

Purpose: To build a new staging system and new prognostic models for MPTB. Methods: We performed a comprehensive analysis of the data from the SEER database. Results: We discussed the characteristics of MPTB by comparing 1085 MPTB cases with 382,718 invasive ductal carcinoma cases. We established a new stage- and age-stratification system for MPTB patients. Furthermore, we built two prognostic models for MPTB patients. The validity of these models was confirmed through multifaceted and multidata verification. Conclusions: Our study provided a staging system and prognostic models for MPTB patients, which can not only help to predict patient outcomes, but also enhance the understanding of the prognostic factors associated with MPTB.

## 1. Introduction

Malignant phyllodes tumor of the breast (MPTB) is a rare fibroepithelial tumor originating in the intralobular and periductal stroma [1]. MPTB account for less than 1% of all primary breast tumors [2]. It consists of two components, an epithelial component and a mesenchymal component, both of which appear to be malignant and progressive [3].

Invasive ductal carcinoma is the most common breast cancer, and has a reliable grading system and a large number of prognostic models. Unlike invasive ductal carcinoma, phyllodes breast cancer lacks a specific patient staging system. The applicability of the staging modalities employed in the case of IDC to MPTB remains a matter of contention, due, in part, to the paucity of comparative research regarding the clinical attributes of these two types of breast cancers. The advent and continual refinement of clinical databases open avenues for a more comprehensive examination of the prognosis of rare neoplasms, such as MPTB. In this study, we used the Surveillance, Epidemiology, and End Results program (SEER), a database providing information on cancer statistics, in order to address these issues.

Several prognostic stratification methods have been developed to analyze prognosis stratification, such as the X-tile method, but most of them can only perform a hierarchical analysis that divides patients into two groups [4]. K-adaptive partitioning for survival data (KAPS), a novel adaptive partitioning algorithm based on the multiway split approach, enables the demarcation of multiple groups [5]. Furthermore, it enables the setting of multiple cross-checks, thus improving the outcome scalability. The widely used Cox proportional hazards regression model, a staple of survival analysis, operates under the assumption of proportional hazards, while the random survival forests (RSF) approach, rooted in machine learning, does not impose such an assumption and can be applied to a broader range of data types [6,7]. These two models have unique characteristics and show their merits under different circumstances [8,9].

In this study, we used public databases to compare the clinical features of MPTB with those of invasive breast cancer, the most common type of breast cancer, and illustrated the necessity of establishing independent staging systems and prognostic models for MPTB. Meanwhile, we used the KAPS algorithm to explore and establish a new stage- and age-stratification system. Our new MPTB T-staging system exhibited a good prognostic stratification ability in both the internal cohort and the external cohort. Furthermore, the Cox proportional hazards regression model and RSF were used to establish independent prognostic models for MPTB, based on our MPTB T stage- and age-stratification system. Both models had a significant predictive power. Finally, we built a user-friendly web app to allow researchers and doctors to access our model.

## 2. Materials and Methods

### 2.1. Data Collection

**Figure 1:**
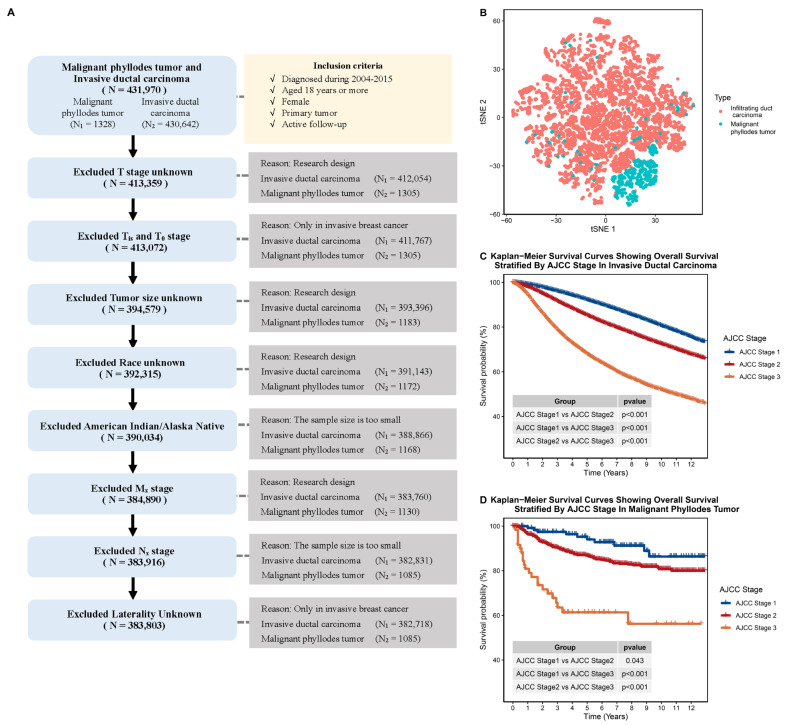
Comparison of clinical characteristics of patients with MPTB and patients with IDC. (**A**) Flow chart of patient selection process in our study. (**B**) The t-SNE plot showing the overall clinical characteristics of patients with MPTB and IDC (based on a sample of 10,000 randomly selected patients). (**C**) Kaplan–Meier plot showing overall survival stratified by AJCC stage in IDC. (**D**) Kaplan–Meier plot showing overall survival stratified by AJCC stage in MPTB.

We collected data on invasive ductal carcinoma (IDC) (ICD-O-3: 8500/3) and malignant phyllodes tumor of the breast (MPTB) (ICD-O-3: 9020/3) cases between 2004 to 2015 from the Surveillance, Epidemiology, and End Results (SEER) database. Only patients with a primary tumor and over 18 years old were considered for further analysis. Male patients, patients in Tis or T0, and patients with unknown laterality were excluded. We also removed American Indian/Alaskan Native cases (N = 4) and Nx Stage (N = 1) cases from the MPTB cohort due to their small number (Figure 1A).

### 2.2. Comparing the Clinical Characteristics of IDC and MPTB

To compare IDC and MPTB, we excluded certain groups of patients who were not present in the MPTB cohort from IDC cohort, such as male patients and those with unknown stage or side (Figure 1A). The Gower distance and t-distributed stochastic neighbor embedding (t-SNE) were used to visualize the data [10,11].

### 2.3. Construction of New T-Stage and Age Groupings

We first divided the patients into two groups according to their region of origin. Patients who originated from 10 randomly selected regions were used as the internal cohort (train set), and those from the remaining 7 regions were used as the external validation cohort (test set).

We applied an improved KAPS algorithm, searching for k = 2–4 split groups, to identify the best tumor size and age segmentation point based on the internal cohort [5].

**Figure 2:**
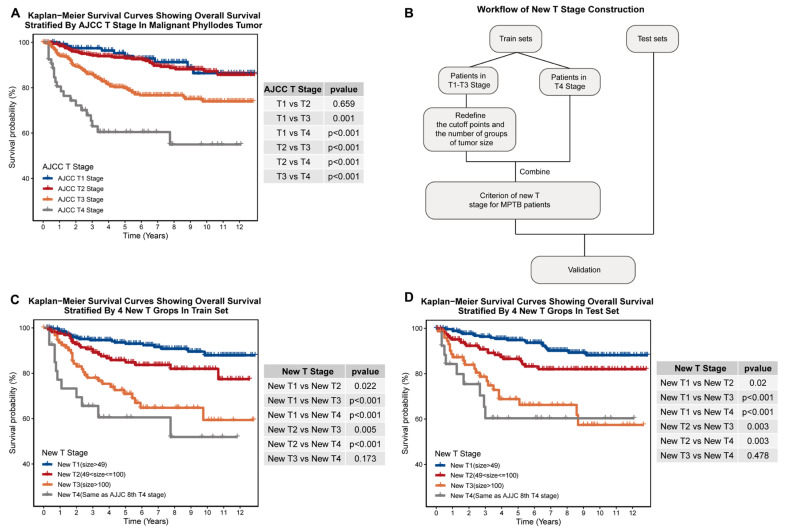
Construction of new T-staging system for MPTB. (**A**) Kaplan–Meier plot showing overall survival stratified by AJCC T stage in MPTB. (**B**) Workflow for the construction of new T-staging system for MPTB. (**C**) Kaplan–Meier plot showing overall survival stratified by new T stage in training set. (**D**) Kaplan–Meier plot showing overall survival stratified by new T stage in test set.

It is noteworthy that patients diagnosed with Stage T4 tumors (tumors with direct extension to the chest wall or skin) were excluded from the tumor size segmentation analysis because of their need for unique surgical management. These patients were considered as a single group in the establishment of our newly proposed staging system, as depicted in Figure 2B [12].

### 2.4. Construction of Prognostic Models

We used multivariate Cox regression and random survival forests (RSF) to construct prognostic models. We included variables with a *p* value less than 0.05 in the univariate Cox regression model and with VIMP less than 0 in RSF as prognostic factors. We used a grid search to find the best parameters for RSF. The combination that resulted in the smallest OOB training error was chosen to be the final set of parameters.

### 2.5. Validation of Prognostic Models

We used receiver operating characteristic (ROC) curves to assess the accuracy of the models [13]. In order to evaluate the proposed model, we carried out decision curve analysis (DCA) on our models [14]. The calibration curves were plotted to assess the calibration of our models [15]. Finally, we built a web-based application to make our new predictive model and new stage estimations available online. The web-based application was built based on the R package “shiny”. All of the analyses were performed using R version 4.1.0 (http://www.r-project.org/, accessed on 1 March 2022).

### 2.6. Statistical Analysis

The Wilcoxon test was utilized to assess the differences among groups of non-normal continuous variables, while the chi-squared test was employed to examine the disparities among the groups of categorical variables. Survival analysis was performed using Kaplan–Meier survival curves, which were compared using the log-rank test [16]. The Benjamini–Hochberg method was used to adjust the *p*-values [17]. All of the analyses were performed using R (version 4.2.0).

## 3. Results

### 3.1. Malignant Phyllodes Tumor of the Breast Has Vastly Different Clinical Characteristics to Invasive Ductal Carcinoma

Whether the AJCC stage can be used to predict the prognosis of MPTB patients is unclear. We assumed that the AJCC TNM staging systems were suitable for MPTB patients. Therefore, we first compared the clinical characteristics of IDC and MPTB patients. Table 1 summarizes the demographic and clinical differences between the two types of breast cancer. Significant variations were found, with IDC patients being older, having a higher rate of lymph node metastasis, and smaller tumor size (*p* < 0.05, Table 1). Conversely, MPTB tumors were more likely to be high-grade, ER-negative, PR-negative, and larger in size, and rarely showed lymph node metastasis (*p* < 0.05, Table 1).

To further demonstrate the differences between MPTB and IDC, we utilized t-SNE to visualize the clinical characteristics of the two cancers. The results, displayed in Figure 1B, showed that MPTB and IDC patients were distinct from one another, indicating substantial differences in clinical characteristics and suggesting that the current staging systems and prognostic models for IDC are not appropriate for MPTB.

Furthermore, we assessed the survival stratification ability of the AJCC TNM staging system in MPTB and IDC. The current TNM staging system effectively distinguished IDC patients with different survival risks (Figure 1C, stage I vs. stage II: *p* < 0.01; stage II vs. stage III: *p* < 0.01; stage I vs. stage III: *p* < 0.01). However, it is not the optimal system for assessing the survival of MPTB patients. The differences in overall survival between stage I and stage II patients were close (Figure 1D, stage I vs. stage II: *p* = 0.043; stage II vs. stage III: *p* < 0.001; stage I vs. stage III: *p* < 0.001).

### 3.2. Our New T-Staging System Exhibits Better Prognosis-Distinguishing Ability Than the AJCC T-Staging System in Both Internal and External Cohorts

The MPTB cohort was dichotomized into two distinct subgroups (see Section 2). The demographic and clinical characteristics of each of these subgroups were recorded and are presented in Table 2.

Given the substantial disparities in tumor size between IDC and MPTB patients (*p* < 0.001; Table 1) and the lack of a clear survival difference between MPTB patients classified as T1 and T2 stage by the existing AJCC T-staging system (T1 vs. T2: adjust *p* = 0.659; Figure 2A), we aimed to develop a new MPTB-specific T-staging system. To ensure practical applicability and personalized therapy and prognosis prediction, we carefully selected the number of groups (k) to be either two, three, or four. The hierarchical levels of the survival analysis revealed significant differences when dividing tumor sizes into two or three groups (k = 2: adjust *p* < 0.05, k = 3: adjust *p* < 0.05; Supplementary Materials, Figure S1). Ultimately, a T-staging system was established based on dividing tumor sizes into four strata, with 49 mm and 100 mm as the threshold values (Figure 2B and Table 3). Our new T-staging system was shown to be more effective for determining MPTB patients’ prognoses than the TNM T-staging system based on significant differences in survival among the different levels of the new system in both the internal and external cohorts (adjust *p* < 0.05; Figure 2C,D).

### 3.3. Construction of Prognostic Age-Stratification System

**Figure 3:**
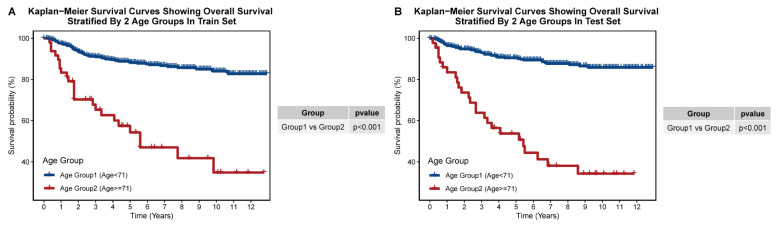
Construction of the age grouping system for MPTB. (**A**) Kaplan–Meier plot showing overall survival stratified by age group in training set. (**B**) Kaplan–Meier plot showing overall survival stratified by age group in the test set.

Similarly, we also established a new risk stratification for patients of different ages using the KAPS algorithm. Statistical differences in survival were only achieved when dividing MPTB patients into two groups (Supplementary Materials, Figure S2). Accordingly, 71 years old was selected as the age threshold, and we created a new age-based risk stratification for patients with MPTB according to this standard (Table 3). Moreover, our age stratification scheme was able to determine prognosis well for patients in both internal and external cohorts (Figure 3A,B; Supplementary Materials).

### 3.4. Construction of MPTB Survival Prognosis Models

Taking into account the application scope and the predictive powers of different models, we used Cox and RSF to establish a prognostic model for MPTB patients. Our findings revealed that several variables, such as laterality, the site of the primary tumor, ER, PR, and radiation therapy, demonstrated no notable influence on the prognosis of MPTB patients. In light of these results, we selected age, new T stage, surgery, chemotherapy, lymph node metastasis, and grade as the final determinants of prognosis, which were then employed in the model’s construction (with *p* < 0.05 in the univariate Cox analysis and VIMP > 0 in RSF; Table 4, Supplementary Materials, Figure S3). To optimize the model’s performance, we executed a preliminary grid search to ascertain the optimal parameters (Supplementary Materials, Table S1). The combination yielding the lowest OOB error was eventually identified and selected (nodesize = 50, nsplit = 1, Mtry = 2).

### 3.5. Calibration and Validation

**Figure 4:**
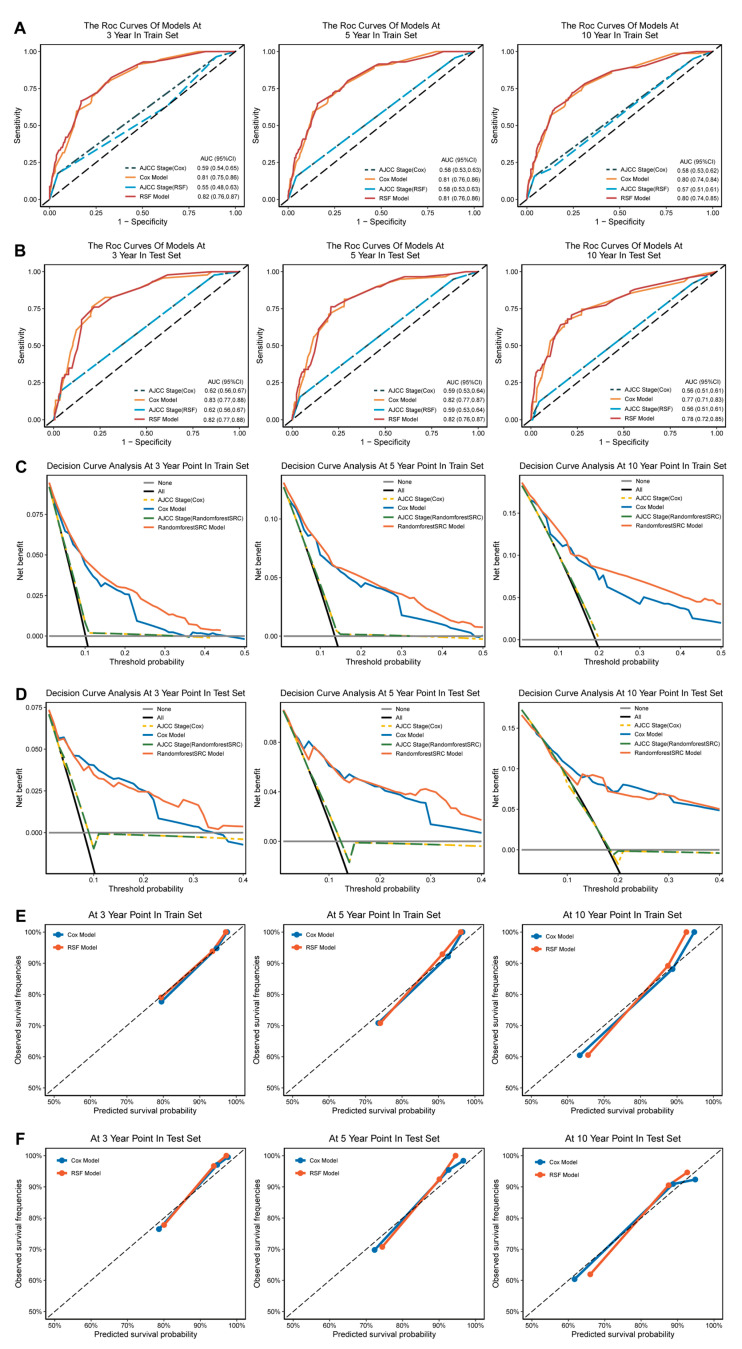
Validation and calibration of models. (**A**) The ROC curves showed that both the Cox model and the RSF model outperformed the AJCC staging system when predicting MPTB patient prognosis at 3, 5, and 10 years in the training set. (**B**) The ROC curves showed that the Cox and RSF models were superior to the AJCC staging system for predicting MPTB patient prognosis at 3, 5, and 10 years in the test set. (**C**) The DCA plots demonstrated that both the Cox and RSF models had a better performance than the AJCC staging system for predicting MPTB patient prognosis at 3, 5, and 10 years in the training set. (**D**) The DCA plots also showed that the Cox and RSF models outperformed the AJCC staging system when predicting MPTB patient prognosis at 3, 5, and 10 years in the test set. (**E**) The calibration curves indicated that the Cox and RSF models accurately predicted MPTB patient prognosis at 3, 5, and 10 years in the training set. (**F**) The calibration curves showed that the Cox and RSF models accurately predicted MPTB patient prognosis at 3, 5, and 10 years in the test set.

The efficacy of our prognostic models was evaluated across multiple dimensions. The AUC values for the Cox model and RRSF model in the training and test sets at 3, 5, and 10 years were higher than 0.75 (Cox model: 0.75–0.8; RSF: 0.76–0.82, Figure 4A,B). The performance of the models was further substantiated by the decision curve analysis (DCA) and calibration curve analysis (CCA), which indicated favorable results in both the training and test sets (Figure 4C–F). These findings reveal the capability of the established prognostic models for accurately predicting the prognoses of patients with malignant pleural mesothelioma at various time points.

We compared the models’ predictive abilities with those of the AJCC staging system. In comparison with our models (both the Cox model and RSF), the AJCC system had a smaller AUC value than that of our models in both the training set and test set at 3, 5, and 10 years (Figure 4A,B). The DCA plot further highlights the superiority of our models, indicating a greater advantage of our models over the traditional AJCC T staging (Figure 4C,D). These results firmly establish the superior prognostic predictive abilities of our models when compared with the AJCC staging system.

### 3.6. Web-Based App Enables Researchers and Clinicians to Easily Share and Use Our New MPTB Staging System and Prognostic Models

**Figure 5:**
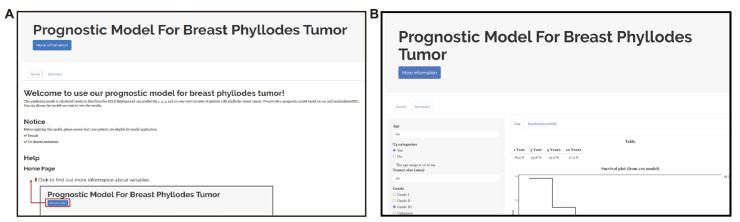
Screenshot of web app. (**A**) Screenshot of the home page of our shiny app. (**B**) Screenshot of the summary page of our shiny app.

In an effort to help researchers and clinicians to learn to use our prognostic model, we created a user-friendly website app (https://prognosticpredictor.shinyapps.io/Phyllodes_tumor/, accessed on 10 February 2023) with a detailed tutorial page providing a walkthrough of the applicable population and prediction processes of the model (Figure 5A,B). Users can apply the Cox or RSF model based on their own needs. Upon entering the clinical characteristics of a new sample, the web app can help to predict the survival probabilities and MPTB stage.

## 4. Discussion

In this study, we conducted a comparison of malignant phyllodes tumor of the breast (MPTB) with invasive ductal carcinoma based on several clinical characteristics, including tumor size and age, highlighting the special characteristics of MPTB and its essential differences from invasive ductal carcinoma. The results further emphasize the point that the staging rules and prognostic models built based on invasive ductal carcinoma may not applied adequately to MPTB. A novel staging system and prognostic models specific to MPTB are needed. Thus, we established a new T-staging system, age grouping, and two prognostic models for MPTB.

We observed huge differences in tumor size and age between MPTB and IDC. Thus, we attempted to build our new MPTB staging system concentrating on tumor size and age, applying a KAPS algorithm with cross-checking and multipoint calculation [5]. Finally, we established a new MPTB age stratification, with 71 years old as the threshold, and a new MPTB T-staging system, grouping tumors with 49 mm and 100 mm thresholds. Better prognostic stratification of patients can benefit clinical practice, as well as improve research and therapies. We anticipate that our new T-staging system will find broader applications in the future, such as serving as a guide for treatment planning. However, it is worth mentioning that due to the paucity of data, we excluded patients with distant metastasis from this study. Further investigations are necessary in order to dissect the features of this cohort.

Unlike other linear dividing algorithms, the KAPS algorithm can not only identify multiple sets of staging points, but also optimize the division through multiple cross-checks, ensuring the scalability of the resulting staging points to a certain extent [5]. In this study, we built two prognosis models for MPTB, a Cox model and a random survival forests model, each with their own characteristics. The Cox model is a widely used assumption-based model for clinical prognosis prediction. Random survival forests, a machine-learning approach known for its ability to deal with data with multiple predictors without assumptions, has also demonstrated potential for prognostic prediction in recent publications. Studies have revealed that random survival forests may achieve a higher predictive power [18,19]; however, these two models displayed equivalent predictive capabilities based on our data. Considering the intuitive nature and characteristics of these two models, we presented both in the paper and evaluated their performance in several ways. We integrated the variable screening results of the two models for the selection of prognostic factors. This integrated approach of prognostic factor selection has also been applied and verified in previous studies.

The main recommended treatment for MPTB is surgery [20]. There is some controversy over whether chemotherapy is effective against MPTB. Our findings showed that chemotherapy is an independent prognostic factor, which is detrimental to the patient’s survival (HR > 1). Several case reports have shown the potential of chemotherapy to be effective in the locoregional control of MPTB [21,22,23,24,25]. Our results showed that there is a huge difference in the expression of key molecules between IDC and MPTB, such as ER and PR (Table 1). Moreover, the results of the Cox regression analysis showed that these key molecules for IDC were not potential prognostic factors for MPTB (Table 3). Considering the obtained outcomes, we hold that it is imperative to engage in further studies with the aim of refining the chemotherapy protocol of MPTB. Nie et al. pointed out that using maraviroc to target CCR5 might provide a clinically feasible solution for MPTB [26]. Our results suggest that attention should be paid to the role of chemotherapy in the treatment of MPTB, and more work is needed to find an appropriate chemotherapy regimen for MPTB.

We also acknowledge that our study has certain limitations. Foremost among these is the exclusion of patients with metastatic disease due to a deficiency of data. Our staging systems and models also need to be verified based on more data to confirm its effectiveness. We could not include more potential prognostic indicators of breast cancer (e.g., HER2) in our calculations due to the limited dataset. Although our model achieved a superior predictive ability in both internal and external cohorts, there is much optimization work to be carried out in the future.

## 5. Conclusions

In conclusion, we established new T-staging and age-grouping systems that can effectively determine the prognostic risk of patients with nonmetastatic MPTB. Meanwhile, we established two prognostic models that performed well based on our new staging system. Multifaceted and multi-data verification demonstrated the survival-prediction capability of our prognostic models.

## Figures and Tables

**Table 1:** Comparison of malignant invasive ductal carcinoma and malignant phyllodes tumor groups.

Characteristics	Overall	Invasive Ductal Carcinoma	Phyllodes Tumor	*p* Value
	(*n* = 383,803)	(*n* = 382,718)	(*n* = 1085)	
Age at diagnosis (years) (median [Q1, Q3])	59.00 [50.00, 69.00]	59.00 [50.00, 69.00]	51.00 [43.00, 60.00]	<0.001
Race (%)				
White	307,371 (80.1)	306,565 (80.1)	806 (74.3)	<0.001
Black	42,359 (11.0)	42,246 (11.0)	113 (10.4)	
Asian or Pacific Islander	34,073 (8.9)	33,907 (8.9)	166 (15.3)	
Marital status (%)				
Never married/Unknown	70,605 (18.4)	70,273 (18.4)	332 (30.6)	<0.001
Ever married	313,198 (81.6)	312,445 (81.6)	753 (69.4)	
Tumor size (median [Q1, Q3])	16.00 [10.00, 26.00]	16.00 [10.00, 26.00]	50.00 [30.00, 90.00]	<0.001
T stage (%)				
T_1_	238,792 (62.2)	238,673 (62.4)	119 (11.0)	<0.001
T_2_	116,232 (30.3)	115,782 (30.3)	450 (41.5)	
T_3_	17,907 (4.7)	17,445 (4.6)	462 (42.6)	
T_4_	10,872 (2.8)	10,818 (2.8)	54 (5.0)	
Lymph node metastasis (%)				
None/Unknown	260,222 (67.8)	259,149 (67.7)	1073 (98.9)	<0.001
Yes	123,581 (32.2)	123,569 (32.3)	12 (1.1)	
8th TNM stage (%)				
I	192,360 (50.1)	192,241 (50.2)	119 (11.0)	<0.001
II	144,838 (37.7)	143,932 (37.6)	906 (83.5)	
III	46,605 (12.1)	46,545 (12.2)	60 (5.5)	
Laterality (%)				
Left—origin of primary	194,348 (50.6)	193,835 (50.6)	513 (47.3)	0.029
Right—origin of primary	189,455 (49.4)	188,883 (49.4)	572 (52.7)	
Site of primary tumor (%)				
Central	19,380 (5.0)	19,304 (5.0)	76 (7.0)	<0.001
UIQ	47,464 (12.4)	47,373 (12.4)	91 (8.4)	
LIQ	22,463 (5.9)	22,435 (5.9)	28 (2.6)	
UOQ	136,855 (35.7)	136,542 (35.7)	313 (28.8)	
LOQ	28,510 (7.4)	28,440 (7.4)	70 (6.5)	
Overlap	129,131 (33.6)	128,624 (33.6)	507 (46.7)	
Grade (%)				
Grade I	75,476 (19.7)	75,328 (19.7)	148 (13.6)	<0.001
Grade II	154,420 (40.2)	154,293 (40.3)	127 (11.7)	
Grade III	143,826 (37.5)	143,557 (37.5)	269 (24.8)	
Unknown	10,081 (2.6)	9540 (2.5)	541 (49.9)	
ER (%)				
Negative	78,786 (20.5)	78,708 (20.6)	78 (7.2)	<0.001
Positive	293,015 (76.3)	292,985 (76.6)	30 (2.8)	
Unknown	12,002 (3.1)	11,025 (2.9)	977 (90.0)	
PR (%)				
Negative	116,220 (30.3)	116,140 (30.3)	80 (7.4)	<0.001
Positive	252,929 (65.9)	252,903 (66.1)	26 (2.4)	
Unknown	14,654 (3.8)	13,675 (3.6)	979 (90.2)	
Radiotherapy (%)				
None/Unknown	180,484 (47.0)	179,636 (46.9)	848 (78.2)	<0.001
Yes	203,319 (53.0)	203,082 (53.1)	237 (21.8)	
Chemotherapy (%)				
None/Unknown	213,584 (55.6)	212,531 (55.5)	1053 (97.1)	<0.001
Yes	170,219 (44.4)	170,187 (44.5)	32 (2.9)	
Surgery (%)				
None/Unknown	12,346 (3.2)	12,336 (3.2)	10 (0.9)	<0.001
Yes	371,457 (96.8)	370,382 (96.8)	1075 (99.1)	

UIQ: upper inner quadrant; LIQ: lower inner quadrant; UOQ: upper outer quadrant; LOQ: lower outer quadrant; Q1: first quartile; Q3: third quartile.

**Table 2:** Demographic features of patients with malignant phyllodes tumor.

Characteristics	Overall	Train	Test	*p* Value
	(*n* = 1085)	(*n* = 553)	(*n* = 532)	
Age at diagnosis (years) (median [Q1, Q3])	51.00 [43.00, 60.00]	51.00 [43.00, 61.00]	52.00 [43.00, 59.00]	0.768
Race (%)				
White	806 (74.3)	419 (75.8)	387 (72.7)	0.478
Black	113 (10.4)	56 (10.1)	57 (10.7)	
Asian or Pacific Islander	166 (15.3)	78 (14.1)	88 (16.5)	
Marital status (%)				
Never married/Unknown	332 (30.6)	184 (33.3)	148 (27.8)	0.06
Ever married	753 (69.4)	369 (66.7)	384 (72.2)	
Tumor size (median [Q1, Q3])	50.00 [30.00, 90.00]	50.00 [31.00, 94.00]	50.00 [30.00, 85.00]	0.225
T stage (%)				
T1	119 (11.0)	53 (9.6)	66 (12.4)	0.501
T2	450 (41.5)	230 (41.6)	220 (41.4)	
T3	462 (42.6)	242 (43.8)	220 (41.4)	
T4	54 (5.0)	28 (5.1)	26 (4.9)	
Lymph node metastasis (%)				
None/Unknown	1073 (98.9)	546 (98.7)	527 (99.1)	0.824
Yes	12 (1.1)	7 (1.3)	5 (0.9)	
8th TNM stage (%)				
I	119 (11.0)	53 (9.6)	66 (12.4)	0.331
II	906 (83.5)	469 (84.8)	437 (82.1)	
III	60 (5.5)	31 (5.6)	29 (5.5)	
Laterality (%)				
Left—origin of primary	513 (47.3)	252 (45.6)	261 (49.1)	0.276
Right—origin of primary	572 (52.7)	301 (54.4)	271 (50.9)	
Site of primary tumor (%)				
Central	76 (7.0)	41 (7.4)	35 (6.6)	0.45
UIQ	91 (8.4)	40 (7.2)	51 (9.6)	
LIQ	28 (2.6)	15 (2.7)	13 (2.4)	
UOQ	313 (28.8)	152 (27.5)	161 (30.3)	
LOQ	70 (6.5)	33 (6.0)	37 (7.0)	
Overlap	507 (46.7)	272 (49.2)	235 (44.2)	
Grade (%)				
Grade I	148 (13.6)	72 (13.0)	76 (14.3)	0.245
Grade II	127 (11.7)	70 (12.7)	57 (10.7)	
Grade III	269 (24.8)	148 (26.8)	121 (22.7)	
Unknown	541 (49.9)	263 (47.6)	278 (52.3)	
ER (%)				
Negative	78 (7.2)	41 (7.4)	37 (7.0)	0.86
Positive	30 (2.8)	14 (2.5)	16 (3.0)	
Unknown	977 (90.0)	498 (90.1)	479 (90.0)	
PR (%)				
Negative	80 (7.4)	42 (7.6)	38 (7.1)	0.854
Positive	26 (2.4)	12 (2.2)	14 (2.6)	
Unknown	979 (90.2)	499 (90.2)	480 (90.2)	
Chemotherapy (%)				
None/Unknown	1053 (97.1)	533 (96.4)	520 (97.7)	0.252
Yes	32 (2.9)	20 (3.6)	12 (2.3)	
Surgery (%)				
None/Unknown	10 (0.9)	4 (0.7)	6 (1.1)	0.704
Yes	1075 (99.1)	549 (99.3)	526 (98.9)	
Radiotherapy (%)				
None/Unknown	848 (78.2)	433 (78.3)	415 (78.0)	0.137
Radiation after surgery	233 (21.5)	116 (21.0)	117 (22.0)	
Radiation prior to surgery	4 (0.4)	4 (0.7)	0 (0.0)	

UIQ: upper inner quadrant; LIQ: lower inner quadrant; UOQ: upper outer quadrant; LOQ: lower outer quadrant. Train set: Connecticut; Detroit (Metropolitan); Greater Georgia; Iowa; Kentucky; Los Angeles; New Mexico; San Francisco—Oakland SMSA; San Jose—Monterey; Utah. Test set: Atlanta (Metropolitan); California excluding SF/SJM/LA; Hawaii; Louisiana; New Jersey; Rural Georgia; Seattle (Puget Sound); Q1: first quartile; Q3: third quartile.

**Table 3:** New staging system for patients with malignant phyllodes tumor.

Classification	Stage	Definition
New T stage	New T stage 1	Tumor < 49 mm in greatest dimension.
	New T stage 2	Tumor ≥ 49 mm but < 100 mm in greatest dimension.
	New T stage 3	Tumor size ≥ 100 mm in greatest dimension.
	New T stage 4	Tumor of any size with direct extension to chest wall or skin.
Age Group	Age Group 1	Age < 71.
	Age Group 2	Age ≥ 71.

**Table 4:** Univariate and multivariate Cox proportional hazards regression of malignant phyllodes tumor.

Characteristics		Univariate		Multivariable	
		HR (95%CI)	*p* Value	HR (95%CI)	*p* Value
Race	White				
	Black	1.500 (0.824, 2.730)	0.185	-	-
	Asian or Pacific Islander	0.908 (0.465, 1.772)	0.776	-	-
Marital status	Never married/Unknown				
	Ever married	1.163 (0.724, 1.867)	0.533	-	-
Lymph node metastasis	None/Unknown				
	Yes	4.367 (1.375, 13.871)	0.012	1.871 (0.564, 6.210)	0.306
Laterality	Left—origin of primary				
	Right—origin of primary	1.054 (0.685, 1.621)	0.813	-	-
Radiotherapy	None/Unknown				
	Yes	1.223 (0.746, 2.006)	0.425	-	-
Chemotherapy	None/Unknown				
	Yes	2.840 (1.309, 6.159)	0.008	2.407 (1.081, 5.363)	0.032
Surgery	None/Unknown				
	Yes	0.109 (0.027, 0.446)	0.002	0.077 (0.018, 0.336)	0.001
Grade	Grade I				
	Grade II	0.704 (0.199, 2.495)	0.586	0.913 (0.253, 3.303)	0.890
	Grade III	3.130 (1.311, 7.472)	0.010	2.599 (1.063, 6.354)	0.036
	Unknown	2.277 (0.966, 5.367)	0.060	1.795 (0.753, 4.282)	0.187
Site of primary tumor	Central				
	UIQ	0.738 (0.241, 2.257)	0.594	-	-
	LIQ	2.046 (0.669, 6.257)	0.209	-	-
	UOQ	0.621 (0.266, 1.451)	0.271	-	-
	LOQ	0.671 (0.202, 2.229)	0.515	-	-
	Overlap	0.982 (0.464, 2.082)	0.963	-	-
New T Stage	New T Stage 1				
	New T Stage 2	1.992 (1.109, 3.579)	0.021	1.844 (1.016, 3.347)	0.044
	New T Stage 3	4.451 (2.524, 7.849)	0.000	4.102 (2.276, 7.393)	0.000
	New T Stage 4	7.187 (3.460, 14.929)	0.000	5.539 (2.595, 11.823)	0.000
Age Group	Age Group 1				
	Age Group 2	5.188 (3.227, 8.341)	0.000	5.173 (3.122, 8.571)	0.000
ER	Negative				
	Positive	0.276 (0.036, 2.142)	0.218	-	-
	Unknown	0.535 (0.283, 1.009)	0.053	-	-
PR	Negative				
	Positive	0.305 (0.039, 2.367)	0.256	-	-
	Unknown	0.540 (0.286, 1.018)	0.057	-	-

HR: hazard ratio; CI: confidence interval.

## Data Availability

The data used in this study were downloaded from the SEER database (https://seer.cancer.gov/, accessed on 15 August 2020). Further information is provided in Section 2.

## Supplementary Materials

The following supporting information can be downloaded at: https://www.mdpi.com/article/10.3390/jcm12051889/s1. Figure S1: Construction of new T-staging system for MPTB patients. (A) Kaplan–Meier plot showing overall survival stratified by three new T stages in training set and test set. (B) Kaplan–Meier plot showing overall survival stratified by four new T stages in training set and test set. (C) Kaplan–Meier plot showing overall survival stratified by five new T stages in training set and test set. Figure S2: Construction of age groups for MPTB patients. (A) Kaplan–Meier plot showing overall survival stratified by two age groups in training set and test set. (B) Kaplan–Meier plot showing overall survival stratified by three age groups in training set and test set. (C) Kaplan–Meier plot showing overall survival stratified by four age groups in training set and test set. Figure S3. Calculation of variable importance (VIMP) for variables. Table S1: Results of the parameter tuning of RSF.
