# Supplementary material for: New Staging System and Prognostic Model for Malignant Phyllodes Tumor Patients without Distant Metastasis: A Development and Validation Study
Source: J Clin Med. 2023 Feb 27;12(5):1889. doi: 10.3390/jcm12051889 (PMC10003404; doi:10.3390/jcm12051889)
Supplement: Supplementary file 1 [file jcm-12-01889-s001.zip › Supplementary Figures.pdf]

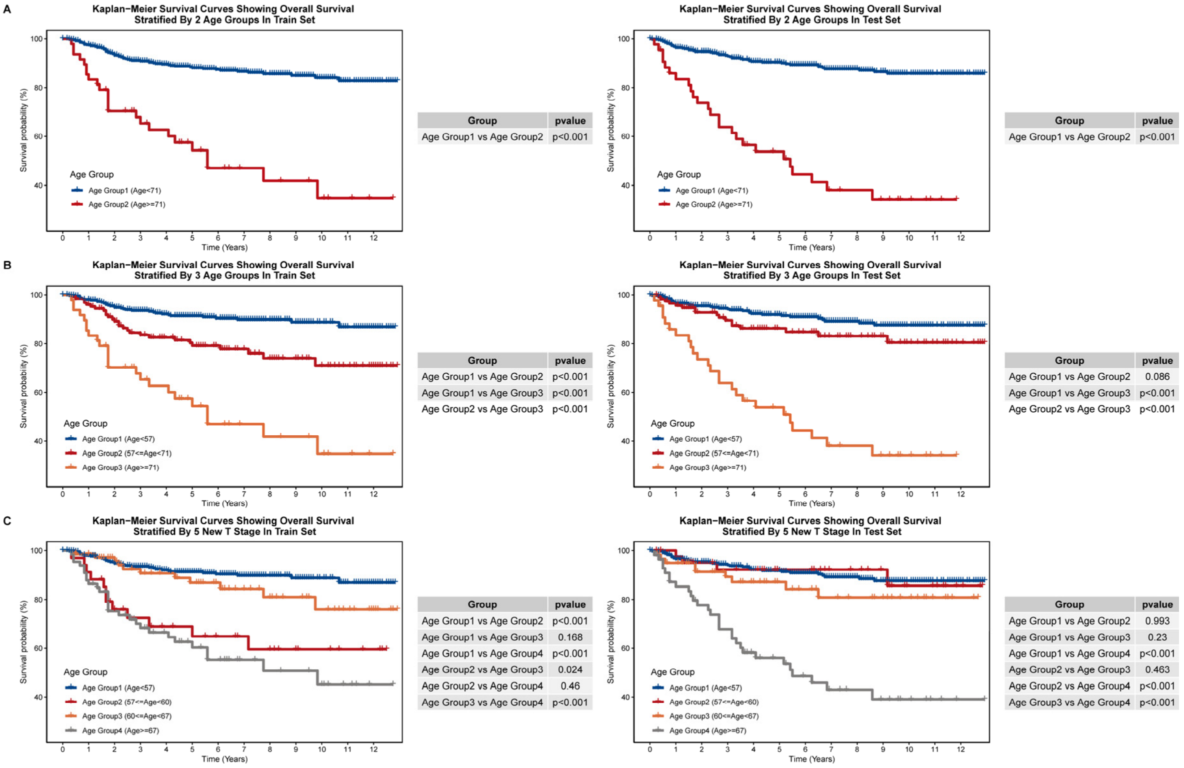

Figure S1: Construction of new T-staging system for MPTB patients. (A) Kaplan–Meier plot showing overall survival stratified by three new T stages in training set and test set. (B) Kaplan–Meier plot showing overall survival stratified by four new T stages in training set and test set. (C) Kaplan–Meier plot showing overall survival stratified by five new T stages in training set and test set.

A

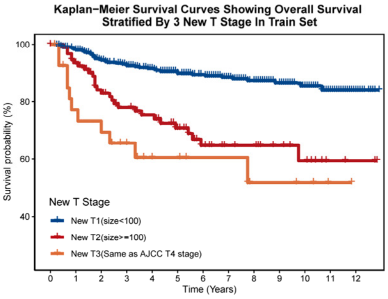

Group	pvalue
New T1 vs New T2	p<0.001
New T1 vs New T4	p<0.001

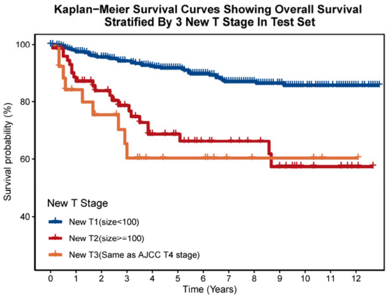

Group	pvalue
New T1 vs New T2	p<0.001
New T1 vs New T4	p<0.001

B

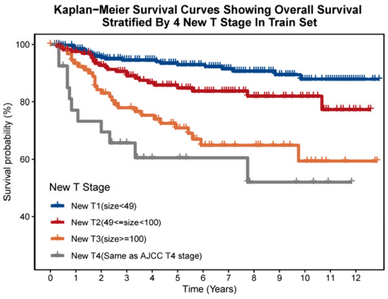

Group	pvalue
New T1 vs New T2	0.022
New T1 vs New T3	p<0.001
New T1 vs New T4	p<0.001
New T2 vs New T3	0.005
New T2 vs New T4	p<0.001

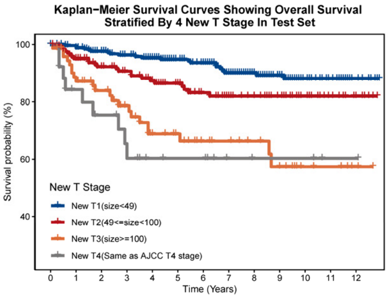

Group	pvalue
New T1 vs New T2	0.02
New T1 vs New T3	p<0.001
New T1 vs New T4	p<0.001
New T2 vs New T3	0.003
New T2 vs New T4	0.003

C

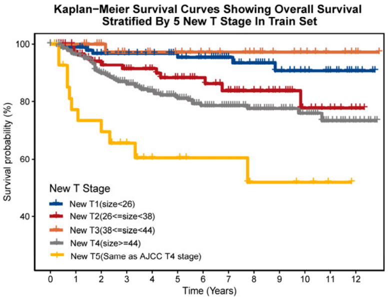

Group	pvalue
New T1 vs New T2	0.064
New T1 vs New T3	0.401
New T1 vs New T4	0.003
New T1 vs New T5	p<0.001
New T2 vs New T3	0.064
New T2 vs New T4	0.281
New T2 vs New T5	0.001
New T3 vs New T4	0.015
New T3 vs New T5	p<0.001

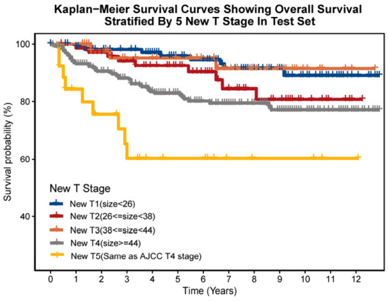

Group	pvalue
New T1 vs New T2	0.222
New T1 vs New T3	0.915
New T1 vs New T4	0.008
New T1 vs New T5	p<0.001
New T2 vs New T3	0.298
New T2 vs New T4	0.23
New T2 vs New T5	0.004
New T3 vs New T4	0.066
New T3 vs New T5	0.002

Figure S2: Construction of age groups for MPTB patients. (A) Kaplan–Meier plot showing overall survival stratified by two age groups in training set and test set. (B) Kaplan–Meier plot showing overall survival stratified by three age groups in training set and test set. (C) Kaplan–Meier plot showing overall survival stratified by four age groups in training set and test set.

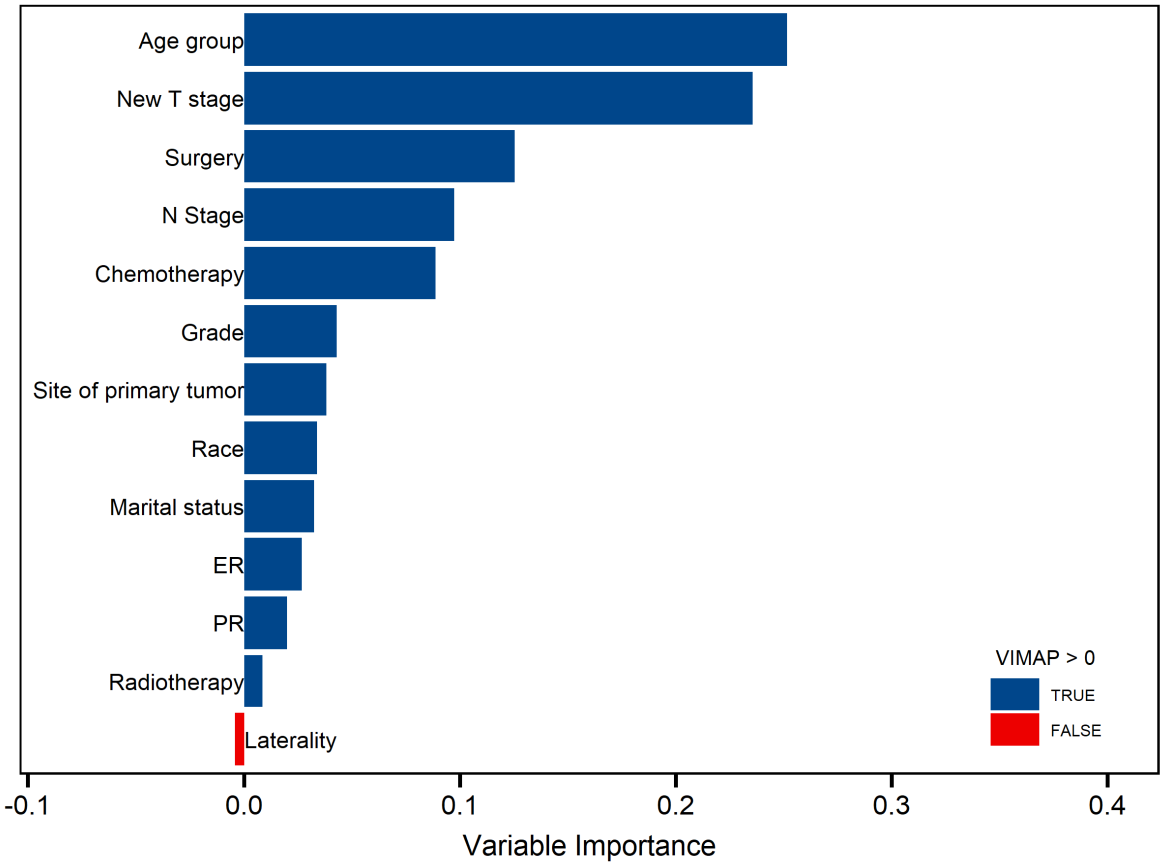

Figure S3: Calculation of variable importance (VIMP) for variables.
